# Vitamin D Deficiency and Its Determinants in Adults: A Sample from Community-Based Settings in the United Arab Emirates

**DOI:** 10.1155/2017/3906306

**Published:** 2017-03-02

**Authors:** Wegdan Bani-issa, Kamal Eldeirawi, Sondos Harfil, Randa Fakhry

**Affiliations:** ^1^Nursing Department, College of Health Sciences, University of Sharjah, P.O. Box 27272, Sharjah, UAE; ^2^Department of Health Systems Science, UIC College of Nursing (M/C 802), 845 South Damen Ave., Rm. 956, Chicago, IL 60612, USA; ^3^Medical Labratory Sciences Department, College of Health Sciences, University of Sharjah, P.O. Box 27272, Sharjah, UAE

## Abstract

*Background.* Vitamin D deficiency (VDD) is a public health concern in adults worldwide. This study aims to explore the extent of VDD and its associated factors among adults in the United Arab Emirates (UAE). *Subjects and Methods.* Quantitative, cross-sectional research was used to assess VDD and its associated factors in 216 adults recruited from randomly selected community-based healthcare settings over a six-month period. Recent values of vitamin D and glycated hemoglobin (HbA1c) were abstracted from medical records, followed by interviews with participants to obtain information on factors related to VDD and other covariates and to measure their heights and weights. *Results.* A total of 74% of participants demonstrated VDD (vitamin D serum level ≤ 30 nmol/L). Emirati participants had higher odds of having VDD compared to non-Emiratis (OR: 2.95; 95% CI: 1.58–5.52), with also significantly increased odds of the condition appearing in older, less educated, and employed adults. Diabetes type 2 (HbA1c ≥ 6.5%), depression, and obesity were significantly associated with an increased likelihood of VDD after accounting for other covariates. *Conclusion.* VDD is a significant problem for UAE adults and requires attention by public health policy makers. Diabetes, obesity, and depression need to be considered when screening for vitamin D.

## 1. Introduction

Vitamin D, 25-hydroxyvitamin D (25(OH) D), is a vital fat-soluble vitamin that regulates calcium homeostasis and is essential for bone and muscle health in people of all ages [1]. Vitamin D is naturally present in some foods and dietary supplements and is produced endogenously when sunlight strikes the skin and stimulates vitamin D synthesis. Serum concentration of 25(OH) D is the best indicator of vitamin D status in humans, with values of less than 30 nmol/L (nmol/L = 0.4 ng/mL) considered to be inadequate for the general health and wellbeing of adults [2].

A plethora of epidemiological and observational studies have demonstrated the correlation between vitamin D and overall human health [3–5]. Studies suggest that sufficient serum vitamin D of more than 30 nmol/L is implicated in preventing cardiovascular disease [6]. An adequate vitamin D serum level has also been reported to enhance the immune system, prevent cancer, and limit its progression [7]. In addition, vitamin D plays a role in the prevention and control of diabetes through improving glycemic index [8], enhancing beta cell function, increasing insulin secretion, and decreasing insulin resistance [9–12].

Evidence is also accumulating for the neurocognitive benefits of vitamin D. A systematic review of 25 cross-sectional studies indicated that lower vitamin D levels were significantly associated with poor cognitive function and dementia [13]. In another systematic review, low vitamin D concentration was found to be associated with the development of depression manifestations [14]. A meta-analysis of 15 randomized controlled trials (RCTs) demonstrated a statistically significant improvement in depression levels with vitamin D supplementation [15]. Researchers concluded that vitamin D supplementation (≥800 I.U. daily) was favorable in the management of depression manifestations [15].

Unfortunately, vitamin D deficiency is increasingly recognized as an endemic condition in adults with global variations in the prevalence of vitamin D deficiency [16]. In the Middle East, vitamin D deficiency is common among adults. In a cross-sectional study of 834 adult men living in Saudi Arabia, 87.8% had a serum 25(OH) D level < 50 nmol/L, with an overall mean of 29 nmol/L [17]. A similar study of 1172 Saudi adult women reported that 80% had serum 25(OH) D levels of <30 nmol/L and about 10% were severely deficient with levels < 12.5 nmol/L [18]. Authors attributed these low levels of vitamin D to poor lifestyle, less physical activity, obesity, and lack of awareness of the importance of vitamin D for health [17, 18]. Levels of vitamin D deficiency are also affected by religious and cultural practices in the region. For example, a study from Jordan found that adult women, who wore complete body coverage and veil, showed high levels of insufficient vitamin D compared to women in Western-style dress [19].

Vitamin D deficiency is a global health problem, with serum levels showing considerable variations across geographical locations, genetic compositions, and demographics of population. It is of utmost importance that the status of vitamin D be assessed in the UAE population. The International Osteoporosis Foundation (IOF) (2016) indicated that residents of the UAE are at a greater risk for bone degeneration, with a vitamin D deficiency rate estimated at 78% as a contributing factor [20]. Adults in the UAE do not receive adequate sun exposure in spite of the fact that the UAE is one of the sunniest countries worldwide, which could place adults at higher risk for vitamin D deficiency [21]. Obesity and diabetes type 2 (as high as 20% in the UAE population) are additional risk factors for vitamin D deficiency [21], and both conditions are attributed to an increase in the economic status, accompanying sedentary lifestyle and reduced physical activity of the population [21]. Regardless of the increased risk for vitamin D deficiency among UAE adults, scarce data are available on the extent of vitamin D deficiency and its associated factors. Hence, this study was undertaken to assess the prevalence of vitamin D deficiency and its associated factors in a sample of adults in the UAE. Potential associated factors were selected based on a review of the relevant literature and included age, nationality (Emirati versus non-Emirati), education level, marital and employment status [22], obesity [21, 22], diabetes type 2 [8–12], and depression manifestations [14, 15].

## 2. Methods

### 2.1. Study Design and Sample

This project was a cross-sectional study with a convenience sample of 260 adults. A list of private and governmental community-based settings in the UAE was prepared, from which a total of 20 clinics was selected randomly to participate in the study. Of these 20 clinics, only eight (in urban and nonurban areas) agreed to be part of the study. These settings were from four emirates in the UAE, including Sharjah (three), Ajman (two), Ras Al Khaimah (two), and Abu Dhabi (one). Community-based healthcare settings are established to provide affordable and accessible healthcare to individuals and their families in the community, by way of diagnosis, prevention, and treatment of general health conditions. The study included participants aged 18–64 years with available data on vitamin D and HbA1c from within the last month. Participants with known debilitating health conditions were excluded from the study. Pregnant women were also excluded because pregnancy alters physiological parameters. To achieve 80% statistical power at a 5% significance level [23], the study sought a minimum of 500 subjects.

### 2.2. Participants' Recruitment

At the participating sites, nurses reviewed medical charts and those whose values of vitamin D and HbA1c from within the last month were available were further evaluated to determine participant eligibility for the study. Then, during their visits with the practitioners, eligible participants were asked by the study research assistants (RAs) to join the study; those who agreed were invited to sign consent forms, complete the general information sheet, have their heights and weights measured, and complete a depression screening scale. Data collection took place from March to August of 2015. The study was approved by the Research Ethics Committee at the PI's institution.

### 2.3. Data Collection

We collected data on the sociodemographic characteristics of participants, including age, gender, nationality, marital and employment status, and education level. During the interview, the participants' heights (with no shoes) and weights (with light clothes) were measured to calculate their body mass index (BMI, kg/m^2^).

Participants were assessed for depression using the Patient Health Questionnaire (PHQ-9), a multipurpose instrument which has been utilized to screen, diagnose, and monitor treatment of depression [24, 25]. The scale is a nine-item tool that provides a quick assessment of depression manifestations within the previous two weeks and can be self-administered by patients and rapidly scored by clinicians [24, 25]. The instrument scores each of the nine DSM-IV items from “0” (not at all) to “3” (nearly every day) [25]. Examples of items include the following: “Over the last two weeks, how often have you been bothered by any of the following problems? Little interest or pleasure in doing things; feeling tired or having little energy; poor appetite or overeating.” The total score ranges from 0 to 27 points with 0–4 indicating minimal depression; 5–9 mild depression; 10–14 moderate depression; 15–19 moderately severe depression; and 20–27 severe depression. Subjects in our study were categorized into depressed (total depression score ≥ 10) versus nondepressed (total depression score < 10) [24, 25].

The Arabic version of the PHQ-9 which was used in the current study was tested in primary care patients in Saudi Arabia and demonstrated adequate validity through criteria standards of the Structured Clinical Interview (SCID-R psychiatric interviews). Agreement between the PHQ-9 and psychiatric interviews was adequate (kappa = 0.65, mean diagnostic agreement = 93%) [26].

### 2.4. Statistics

Statistical analysis was performed using Statistical Analysis Software (SAS). Descriptive statistics, including frequencies and percentages for categorical variables, were generated. Subjects were categorized based on their HbA1c levels in accordance with the American Diabetes Association (ADA) (2016) criteria as having diabetes (HbA1c ≥ 6.5%), or not having the disease (HbA1c < 6.5%) [27]. Similarly, on the basis of the serum vitamin D values, subjects were clustered into deficient (≤30 nmol/L) and normal (>30 nmol/L) [1]. For depression values, participants were categorized into depressed (total score ≥ 10) and not depressed (total score < 10) [24]. We also conducted bivariate correlations between vitamin D deficiency and all study correlates.

Multiple logistic regression analysis was used to determine independent associated factors of vitamin D deficiency in this population. The model included age (grouped as 23–39, 40–44, and ≥56 years); gender; nationality (Emirati versus non-Emirati); employment status (employed versus not employed); BMI, categorized as <25 (normal), 25–29 (overweight), and ≥30 (obese); vitamin D deficiency (deficient versus normal); depression (depressed versus not depressed); and diabetes status, based on HbA1c (diabetic versus nondiabetic). A *p* value of ≤0.5 was considered as statistically significant.

## 3. Results

### 3.1. General Demographic and Clinical Characteristics of Participants

Of the 380 charts with values of vitamin D and HbA1c available at selected sites, 120 were excluded for not meeting the study criteria. Among the remaining 260 charts that met the study criteria, 216 subjects agreed to be part of the study.

As shown in Table 1, approximately 63% of the participants were females, 68% Emiratis, 73% married, 11% single, 17% widowed or divorced, and over 68% were 40 years of age or older. Around 63% had secondary school or less of education, and nearly 44% were employed. Values of BMI indicated that 36% of subjects were overweight and 44% were obese. A total of 61% of subjects in our sample had diabetes mellitus type 2 based on their HbA1c levels (6.5% HbA1c or higher is indicative of diabetes). With regard to depression, 62% reported depression manifestations with a total score of ≥10 on PHQ-9.

### 3.2. Vitamin D Levels in Study Population and Its Associated Factors

Only 26% of the total subjects had sufficient vitamin D levels whereas 74% had vitamin D deficiency. In bivariate analyses, the prevalence of vitamin D deficiency was significantly higher in Emirati compared to non-Emirati adults (OR: 2.95; 95% CI: 1.58–5.52, *p* = 0.0006) and was positively and significantly associated with age. Almost 96% of participants aged 56 years or older had vitamin D deficiency compared to participants 40–55 (73.53%) and 23–39 years of age (57.35%). Less educated, as well as unemployed participants, had a significantly higher prevalence of vitamin D deficiency than the more educated and employed participants. Vitamin D deficiency was significantly higher in participants with a depression score of ≥10 than those with a depression score of <10 (92.5% versus 41.5%, *p* < 0.0001, resp.).

There was a positive and significant association between BMI and vitamin D deficiency. The likelihood of vitamin D deficiency in participants with a BMI of 30 or higher was over five times than that observed in their peers with a BMI < 25. Finally, the prevalence of vitamin D deficiency was significantly greater in those with diabetes type 2 than their counterparts free of the disease (92.4% versus 42.9%, *p* < 0.0001).

In multiple logistic regression analyses (Table 2), the factors that remained significantly associated with vitamin D deficiency were depression, diabetes, and obesity whereas age, gender, and nationality were not. The odds of vitamin D deficiency in participants with depression were over 31 times than those observed in nondepressed participants (95% CI: 9.2–105.4). Similarly, participants with diabetes had significantly increased (almost 25-fold) odds of having vitamin D deficiency (95% CI: 7.29–84.99) compared with those without diabetes. With regard to BMI, participants with a BMI > 30 had over six times the odds of having vitamin D deficiency than those with a BMI < 25, who were not significantly different from those in the 25–29.9 BMI range with regard to the likelihood of having vitamin D deficiency.

## 4. Discussion

Our study explored the extent of vitamin D deficiency and its associated factors in UAE adults. We found that 73% of adults had vitamin D deficiency, adding to the evidence that vitamin D is a public health concern and warrants further attention [2–5]. Prevalence figures for vitamin D deficiency in our study population are comparable to those reported in other countries in the Arabian Peninsula. For example, in Kuwaiti adults, both men and women, approximately 56%, had vitamin D inadequacy (25(OH) D = 12–19.9 ng/mL) and 27% had vitamin D deficiency [28]. Therefore, the extent of vitamin D deficiency reported among adults in our study gave more support to the claim that the Middle East region, including the UAE, has the highest levels of vitamin D deficiency compared to other parts of the world—regardless of the abundance of sunshine in the region [21].

The current study highlighted some important population-based factors that are significantly associated with levels of vitamin D deficiency and need to be considered by practitioners working in public health settings. Demographic factors have long been a source of interest in the epidemiology of vitamin D deficiency [19, 21, 22]. Interestingly, our study found that vitamin D levels were significantly lower in Emirati as compared to non-Emirati adults. Such a contrast might be attributed to rapid economic development in the country which has resulted in dramatic changes in Emiratis' lifestyle, their adoption of Western-style luxuries, and the heightened use of technology contributing to less physical activity and much more time spent indoors [21]. Many of the demographic factors used in the current study, including marital status, education level, and employment status, were found to be significant factors associated with vitamin D deficiency; age was however the most significant. Strong support for this positive correlation between vitamin D deficiency and age has been reported across epidemiological studies [22]. Such results were expected, since children in the Middle East, compared to other age groups, spend more time outside, which contributes to their greater sun exposure and, therefore, higher levels of vitamin D compared to adults [19]. Cultural practices related to lifestyle need to be investigated as this might impact levels of vitamin D in adults.

As for gender, most studies have reported that women had lower levels of vitamin D compared to men due to high body fat composition and the effect of childbearing [29]. Our results did not support such a claim—both men and women exhibited similar rates of vitamin D deficiency. This finding was in contrast to other studies of adults in the same region with great similarities in lifestyle, type of food consumed, and weather. For instance, being a female was found to be an independent predictor for vitamin D deficiency or insufficiency among Saudi adults (odds ratio [OR]: 2.992; 95% confidence intervals [CI] 2.069–4.327) [30]. Such variance in the associated factors with vitamin D deficiency suggests the importance of adopting individualized treatment plans for each subgroup of the population that considers not only age and gender, but also education level, employment, marital status, and the interaction among these variables as they may impact vitamin D levels in adults [15, 22].

We found significant bivariate associations of obesity, diabetes type 2, and depression with vitamin D deficiency. These associations were independent of each other and persisted after controlling for other covariates. Obese adults (those with BMI ≥ 30) in our study were found to have high levels of vitamin D deficiency. This finding is consistent with results from previous studies, indicating the inverse relationship between obesity and lower levels of vitamin D [21, 22].

Adults with diabetes type 2 (HbA1c ≥ 6.5) in our sample exhibited lower levels of vitamin D compared to those without diabetes. Robust evidence exists in the literature on the relationship between vitamin D and risk and treatment for diabetes type 2 [8–12]. Further intervention studies are needed to examine if causation exists between different doses of vitamin D supplements and prevention and improvement in glycemic control in subjects with diabetes type 2 [12].

It is important to note that obesity and diabetes type 2 are highly correlated and were reported to be main risk factors for vitamin D deficiency in cross-sectional [24] and RCT studies [10]. Until further confirmatory causation studies are available, it is recommended that greater global attention be devoted to changes in adult lifestyles that will promote in decreasing their body weights and, thus, the risks for developing diabetes type 2 which, in turn, may affect their vitamin D levels. The reported associations between obesity, diabetes type 2, and vitamin D deficiency warrant further examination to evaluate the nature of any causal relationship between these conditions and to guide practitioners in their treatment and management.

As stated above, depression was found to be significantly correlated with vitamin D deficiency, which is congruent with other studies [14]. In a meta-analysis of 15 RCTs searching for evidence of the relationship between vitamin D deficiency and depression, Spedding [15] reported that vitamin D supplementation could have a therapeutic effect in the treatment of depression. Unfortunately, in the UAE, little attention is paid to screening for depression in clinical practice. This could leave the majority of people undiagnosed and more prone to health implications. In fact, depression is not commonly accepted as a subject for disclosure in the UAE. More RCTs are needed to determine if vitamin D supplementation could be beneficial in treating depression in adults.

## 5. Conclusion

Our study found that vitamin D deficiency represents a significant problem for adults in the UAE. Alerting health organizations or governmental bodies at national, regional, and international levels to tackle the problem of vitamin D deficiency and to enhance public awareness about the need for maintaining sufficient vitamin D levels is one of the most important implications of our study. All adults need to be screened for vitamin D levels notwithstanding the escalating cost of the test. In the UAE, the cost of vitamin D testing should be covered by medical insurance policies, as is any other medical procedure. Individualized treatment plans in accordance with current evidence-based practice guidelines for treatment of low vitamin D levels are recommended by the IOM [2]. Normal ranges and treatment recommendations for vitamin D that are applicable in Europe or the US may not work equally well with other cultures, with different lifestyles, social norms, amount of sun exposure, food preferences, genetic composition, clothing, and health status. Individualized treatment plans should consider demographic, cultural practices, and clinical characteristics of adults before treatment for vitamin D deficiency is initiated. National policies may include promotion of dietary recommendations, food fortification, vitamin D supplementation, and judicious sun exposure but should also take into account national, cultural, and dietary habits of people in relation to vitamin D intake and consumption [19]. Greater awareness campaigns are needed to be implemented to increase awareness of the public about sources, screening, and importance of vitamin D for health, especially in the diabetic population.

Further clinical studies are needed on the temporal link between vitamin D and obesity, diabetes type 2, and depression. To date, there are numerous publications on the nonskeletal benefits of vitamin D, but years are required for the results of large, ongoing RCTs examining the nonskeletal benefits of vitamin D to become available for use (IOM) [2]. Further studies should aim to determine cut-off points for serum vitamin D deficiency based on population characteristics to avoid problems of both under- and overtreatment. Our study has limitations in the small sample size and the nature of uncontrolled condition of data collection. There is a need for more clinical and epidemiological studies to be conducted in the UAE about vitamin D deficiency using larger sample sizes and more controlled conditions. Further, it is important to mention that this was a cross-sectional study and hence causal relationships cannot be inferred and that prospective studies to identify determinates are needed.

## Figures and Tables

**Table 1:** Characteristics of the sample and bivariate associations of vitamin D deficiency with covariates (*n* = 216).

Variable	Total *N* (%)	^∗^% with vitamin D deficiency	^∗∗^ *p* value	Odds ratio (OR)	Lower limit	Upper limit
Gender						
Female	136 (62.96)	69.85	0.15	0.625	0.327	1.196
Male	80 (37.04)	78.75				
Nationality						
Emirati	147 (68.06)	80.27	0.0006	2.950	1.575	5.524
Non-Emirati	69 (31.94)	57.97				
Marital status						
Married	157 (72.69)	71.97	0.019	1.976	0.807	4.835
Single	23 (10.65)	56.52		1		
Widow/divorced	36 (16.67)	88.89		6.152	1.633	23.182
Age group (years)						
23–39	68 (31.48)	57.35	<0.0001	1		
40–55	102 (47.22)	73.53		2.066	1.077	3.963
56+	46 (21.30)	95.65		6.350	3.663	72.983
Educational level						
Secondary or less	137 (63.43)	85.40	<0.0001	5.422	2.837	10.364
University	79 (36.57)	51.90		1		
Employment						
Yes	95 (43.98)	55.79	<0.0001	1		
No	121 (56.02)	86.78		5.200	2.678	10.099
Depression levels						
Depression (score ≥ 10)	134 (62.04)	92.54	<0.0001	17.51	8.03	38.18
No depression (score < 10)	82 (37.96)	41.46				
Obesity (body mass index)						
24.99 or less	44 (20.37)	56.82	0.0001	1		
25–29.99	78 (36.11)	65.38		1.436	0.673	0.061
30+	94 (43.52)	87.23		5.193	2.219	12.152
Diabetes type 2						
No diabetes, HbA1c < 6.5	84 (38.89)	42.86	<0.0001	1		
Diabetes, HbA1c > 6.5%	132 (61.11)	92.42		16.267	7.486	35.348

^∗^Cut-off value for vitamin D deficiency is serum (25(OH) D) of ≤31 nmol/L. ^∗∗^*p* value significant at ≤0.05.

**Table 2:** Predictors of vitamin D deficiency based on multiple logistic regression analysis (*n* = 216).

Variable	Odds ratio (OR)	Lower limit	Upper limit	^∗^ *p* value
Age (years)				
Age group 1				
40–55 versus 23–39	0.849	0.269	2.685	0.7806
Age group 2				
56+ versus 23–39	2.812	0.388	20.380	0.3062
Gender				
Female versus male	0.444	0.141	1.400	0.1658
Nationality				
Emirati versus non-Emirati	1.224	0.350	4.286	0.7515
Depression				
Depressed (score > 10) versus nondepressed (score < 10)	31.134	9.197	105.395	<0.0001
Diabetes type 2				
Diabetes (HbA1c ≥ 6.5) versus no diabetes (HbA1c < 6.5)	24.889	7.289	84.989	<0.0001
Obesity (BMI)				
BMI (25–29.99 versus 24.99 or less)	1.064	0.288	3.924	0.9262
BMI (30+ versus 24.99 or less)	6.552	1.516	28.318	0.0118
Employment				
Employed versus nonemployed	3.597	0.986	13.126	0.0536

^∗^ *p* value significant at ≤0.05.
